# Synthesis of graphene/DPA composite for determination of nicotine in tobacco products

**DOI:** 10.1038/s41598-017-13716-2

**Published:** 2017-10-30

**Authors:** Yanqiu Jing, Baohua Yu, Penghui Li, Bin Xiong, Yuyuan Cheng, Yaoguang Li, Chunguang Li, Xianyi Xiao, Mengqi Chen, Liangyuan Chen, Yu Zhang, Mingqin Zhao, Chuance Cheng

**Affiliations:** 1 College of Tobacco Science, Henan Agricultural University, Zhengzhou, Henan province China; 2 Economics and Management College, National Tobacco Cultivation and Physiology and Biochemistry Research Centre, Henan Agricultural University, Zhengzhou, China; 3 Technology Center of Hubei China Tobacco Industry Co, Ltd., Wuhan, Hubei Province China; 4 Nanyang Branch of Henan Tobacco Corporation, Nanyang, Henan Province China; 5 Technology Center of Henan China Tobacco Industrial Co, Ltd., Zhengzhou, Henan Province China; 6 Ganzhou Branch of Jiangxi Tobacco Corporation, Ganzhou, Jiangxi Province China; 7 Key Laboratory of Tobacco Processing Morphology Research in Tobacco Industry of CNTC, Zhengzhou, Henan Province China; 8 School of Geographical Science and Tourism, Meizhou Jiaying University, Meisong Avenue, Meizhou, 514015 China

## Abstract

In this contribution, the azo dye (E)-1-(4-((4-(phenylamino)phenyl)diazenyl) phenyl)ethanone (DPA) was combined with reduced graphene oxide (RGO) for the electrochemical modification of a pencil graphite electrode (RGO/DPA/PGE) surface. A series of electrochemical measurements were used for the characterization of the modified electrode surfaces. At the modified electrode, nicotine was irreversibly reduced. An obvious increase was observed in the reductive peak current of nicotine at the modified electrode, indicating the capability of the RGO/DPA composite to increase the electron transfer rate. The current was found proportional to the nicotine concentration in a range of 31 to 1900 μM, and the limit of detection (LOD) was calculated as 7.6 μM.

## Introduction

Nicotine, belonging to pyridine derivative alkaloid with fatal toxicity, is regarded as one of the most significant ingredients of cigarette and tobacco^1^. The dibasic nature of nicotine results from the two protonation sites at the pyrrolidine and pyridine nitrogens^2^. Nicotine exists in three forms according to different pH values^3^, with their percentages described in previous literatures^4^. The environmental acidity or alkalinity of nicotine molecules is exceptionally important in controlling their physical and chemical properties^5,6^. Aqueous solutions from conventional cigarettes typically have a neutral or slightly acidic pH (5–6), and basically all nicotine in the filler material exists primarily as a monoprotonated salt with the strong ionic forces minimizing evaporative base nicotine loss. The nicotine molecular exists almost exclusively as a diprotonated species under highly acidic aqueous conditions (ph < 3). However, the diprotonated form is rather insignificant, since relatively low pH conditions are normally absent in tobacco. And the nicotine in free base form could be converted into the gas phase through volatilization^7,8^. Therefore, weak acidic condition is optimum for the analysis of total nicotine in tobacco products.

The analysis of nicotine in different sample materials, including breast milk, plasma and cigarette was of vital importance in reflecting the content level of tobacco and to further assess the corresponding physiological and pathological toxicity^9–11^. Substantial attention has been paid to the analysis of nicotine, such as spectrophotometry^12^, chromatography^13^ and amperometric assay^14^. Among these techniques, several strategies, including spectrophotometry, need isolation of nicotine from the sample matrix, which result in substantial analyte loss. For the high performance liquid chromatography analysis, toxic organic solvents are needed in large amount, and this process is time-consuming (tens of minutes). In recent years, the analysis of nicotine has been performed using some biosensors, where the enzyme activity of acetylcholinesterase was inhabitated, thus catalysing the hydrolysis ofneurotransmitter acetylcholine^15,16^. Due to the high cost of enzymes, the use of this techqnique for the determination of nicotine under any real system should involve the consideration of cost. Furthermore, the analysis would be inconvenient due to the easy distortion or denaturalization of enzymes. Hence it is of vital significance to develop a novel analytical technique with desirable convenience, low cost and rapidness. Solid electrode – involved electroanalysis is one of the optimum strategies for the detection of species in solution, since this method is low-cost, easily used, and reliable. Nevertheless, the application of the solid-electrodes to direct electrochemical measurement has been rarely reported in the analysis of nicotine^17,18^.

Considering the low cost, excellent electrical conductivity, and large surface area, graphene is an ideal nano-material for electrochemistry^19^. Due to the synergetic effect of the electrocatalytic activity to improve the sensor sensitivity, graphene-based electrodes have gained extensive application for sensing platforms during the preparation of electrochemical sensors and biosensors^20^. Graphene oxide (GO) and reduced graphene oxide (RGO) are most commonly used graphene-based electrodes, due to their high surface area as graphene, and great numbers of oxygen-containing functional groups as possible precursors for nanocomposite fabrication^21,22^. GO could be electrochemically reduced into RGO using a one-step electrodeposition route through the direct electrodeposition of graphene films from GO dispersions. The conventional chemical techniques suffer disadvantages including lack of control of film thickness, contamination of the resulting product, involvement of toxic chemicals, etc., whereas electrochemical reduction of GO to graphene is a rather rapid and eco-friendly strategy^23^.

On the other hand, azobenzene functionalized carbon nanotubes^24^, graphene oxide^25^, and graphene^26^ have been proposed to have exhibited modulated conductance upon UV irradiation. Furthermore, these materials have been investigated for the determination of hydrogen peroxide and sulfide^27^. Azo group has been applied to the indirect determination of non-electroactive metals, with its significance embodied in the dyestuff industry^28,29^. In this study, the combination of DPA and GO contributed to the successful fabrication of an RGO/DPA modified PGE via electropolymerization. And the determination of nicotine was based on this electrode. Moreover, our developed electrode was highly sensitive, selective and stable in the direct detection of nicotine in tobacco samples.

## Experiments

### Chemicals and materials

Nicotine standard sample (98% purity) and tobaccos were obtained from Tobacoo Research Institute of Hubei Province. GO (2.00 mg/mL) was commercially available in Nanograf. For GO suspension, GO sheets were dispersed in in acetate buffer solution. DPA and tetrabutylammonium perchlorate salt were dissolved by dichloromethane. All chemicals were used as received without additional purification. Deionized double-distilled water was used throughout the preparation of all aqueous solutions.

### PGE Modification

A simple two-step electrochemical route was used to modify the PGE. Specifically, PGE was immersed into 0.10 M tertbutylammonium perchlorate (TBAP)/dichloromethane solution containing 0.63 M DPA. Over the surface of PGE, the electropolymerization of DPA was performed using cyclic voltammetry (CV) measurement between +1.50 V and −1.50 V (versus Ag/AgCl) at 100 mV/s with various numbers of cycles. The polymer thickness was determined as 30 cycles on the as-prepared PGE. Graphene modification was performed over the DPA/PGE through electropolymerization in GO solution (2.00 mg/mL) between +0.50 V and −1.50 V (versus Ag/AgCl) with various numbers of cycles. The accumulation amount of the polymer was determined as 10 cycles on DPA/PGE.

### Characterization

Electrochemical analyses were carried out on a CHI 660 electrochemical workstation using standard three-electrode geometry, where the working, reference, and auxiliary electrodes were DPA/PGE, a saturated calomel electrode (SCE), and a platinum wire, respectively. 0.1 M Na_2_C_2_O_4_ (pH 4.5) supporting electrolyte solution was used throughout unless otherwise stated. Voltammetric curves were obtained after baseline correction, with data recorded at ambient temperature.

### Cigarette sample preparation

Commercial cigarettes were purchased in a local cigarettes shop. Tobacco was obtained after peeling off the filter and rolling paper from ten cigarettes of each brand, and then mixed together before drying in an oven. 1 g tobacco powder was introduced to a 50 mL beaker and then mixed with deionized water (20 mL). Afterwards this receptacle was capped. This was followed by the sonication of the as-prepared mixture for 0.5 h under ultrasonic water bath at ambient temperature. Finally a clear filtrate was obtained after filtering the slurry, and used as the test sample^30^.

### Results and discussion

**Figure 1:**
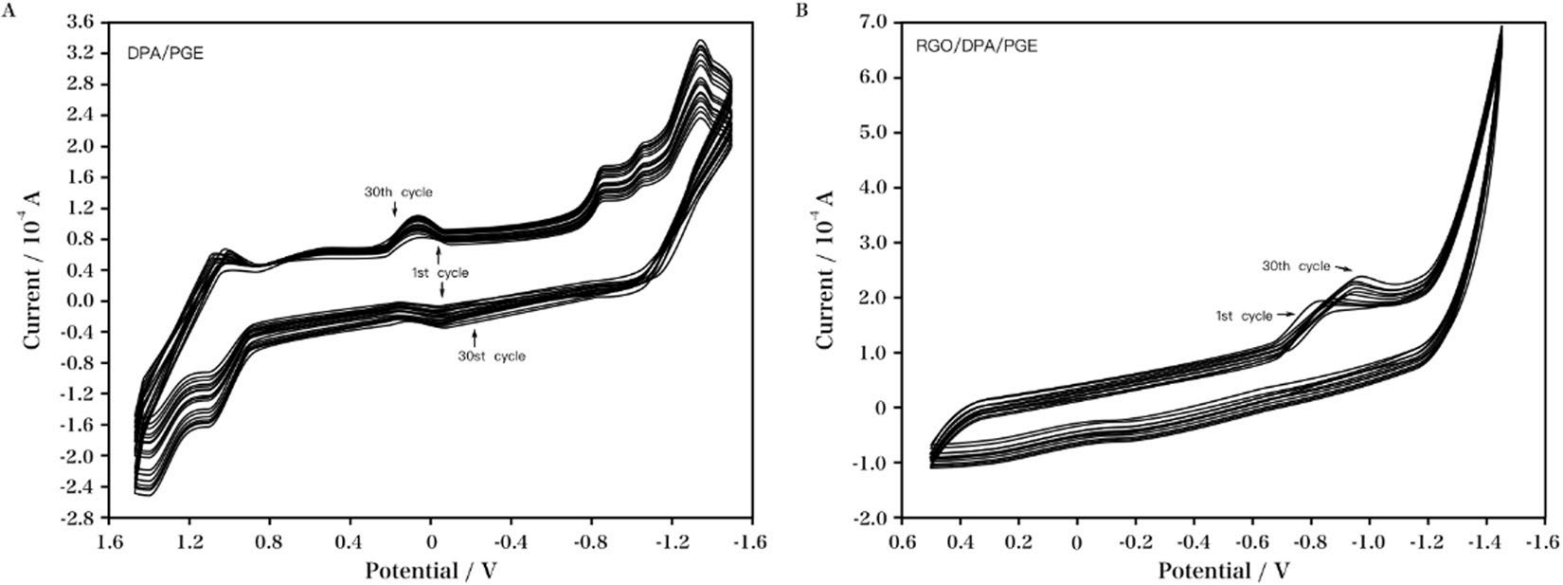
CVs of (**A**) DPA/PGE at a potential range of +1.50 V to −1.50 V in 0.10 M TBAP/dichloromethane solution containing 0.63 mM DPA. Scan rate: 100 mV/s; (**B**) RGO/DPA/PGE at a potential range of +0.50 V to −1.50 V in 2.00 mg/mL GO solution. Scan rate: 50.00 mV/s.

DPA was electropolymerized on the PGE surface using CV measurement at a potential range of +1.50 V to −1.50 V (versus Ag/AgCl) in 0.10 M TBAP/dichloromethane solution containing 0.63 mM DPA, and the scan rate was 100 mV/s. And Fig. 1A showed the corresponding CVs. In the cathodic scan, five peaks were recorded. In the reverse cycle, all the peaks had corresponding oxidation peaks. The initial two cathodic peaks resulted from the reduction of the azo (–N=N–) group to hydrazo (–NH–NH–) group^31^. A steady increase in the current of the above peaks was observed with each scan, indicating that a conducting polymer film grew on the electrode. The CV response to the formation of RGO/DPA at the PGE was shown in Fig. 1B. An obvious reduction current peak was observed in the CV profile of the exfoliated GO (+0.50 to −1.50 V), suggesting the reduction of the surface oxygen groups of GO at *ca*. −1.00 V (starting potential: *ca*. −0.70 V). Hereby the DPA/PGE and RGO/DPA/PGE were obtained as the test electrodes in this study.

**Figure 2:**
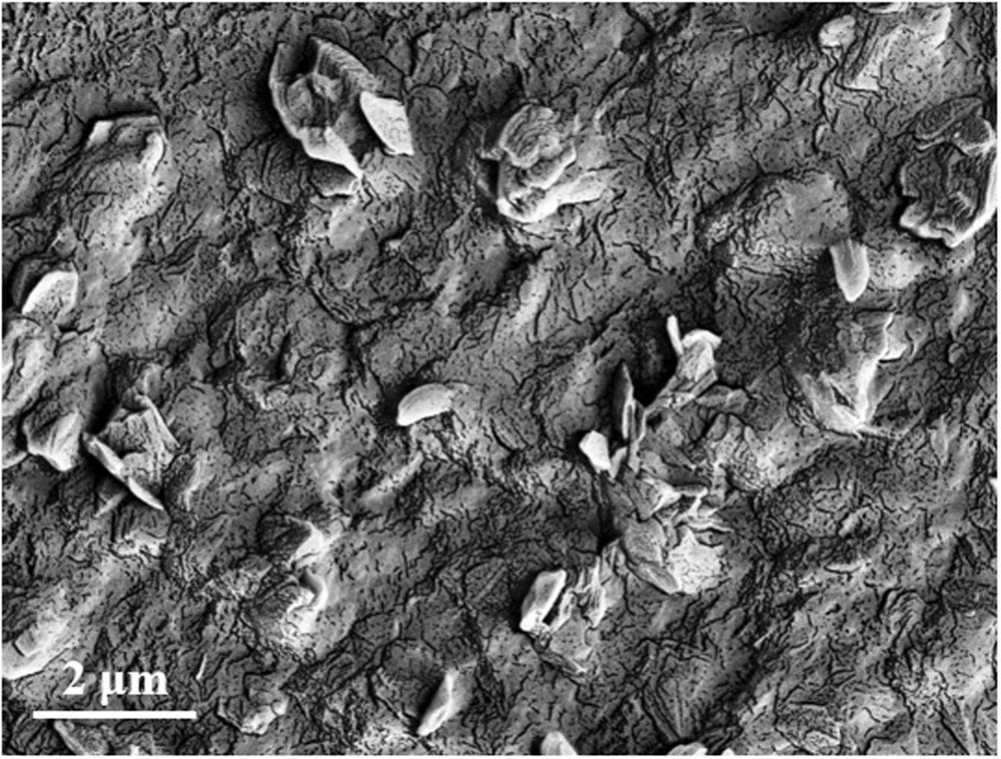
SEM image of the RGO/DPA/PGE.

The surface morphology of the RGO/DPA/PGE was characterized using SEM and shown in Fig. 2. It can be seen that the flake shaped RGO sheets were embedded in the electro-polymerized DPA. Many small cracks could be observed on the composite surface, which enables the target diffusion during the electrochemical reaction.

**Figure 3:**
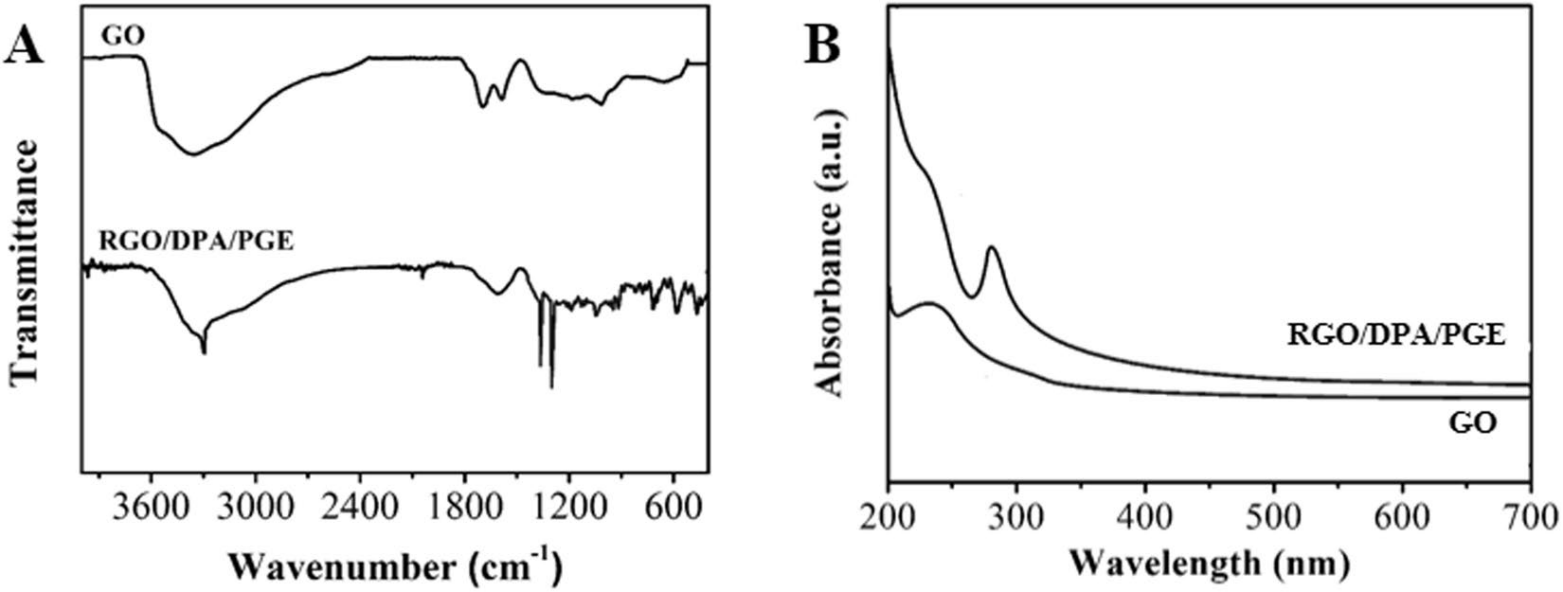
(**A**) FTIR spectra and (**B**) UV-vis spectra of GO and RGO/DPA.

The surface chemical status of the GO and RGO/DPA have been investigated using a FTIR technique. As shown in the Fig. 3A, The IR spectrum of GO presents peaks at 1732, 1622, 1395 and 1049 cm^−1^, which are assigned to the C**=**O stretching of COOH groups, C**=**O stretching vibration, C**—**OH stretching vibration and C**—**O vibrations from alkoxy groups^32–34^, respectively. After CV reduction, the intensity of these peaks becomes much less, indicating that the amount of oxygen-containing groups at the surface of GO is greatly reduced. Moreover, N-H bendings at 1403–1458 cm^−1^ were designated to the hydrazo group in the structure of DPA, suggesting the successful formation of RGO/DPA composite. Figure 3B shows the UV-Vis spectra of water dispersion of GO and RGO/DPA. The GO spectrum exhibits a characteristic absorption peak at 231 nm corresponding to the π → π* transition of aromatic C = C bonds. After CV reudction, this peak shifts from 231 to 288 nm, giving further evidence that most GO has been reduced to RGO^35^.

**Figure 4:**
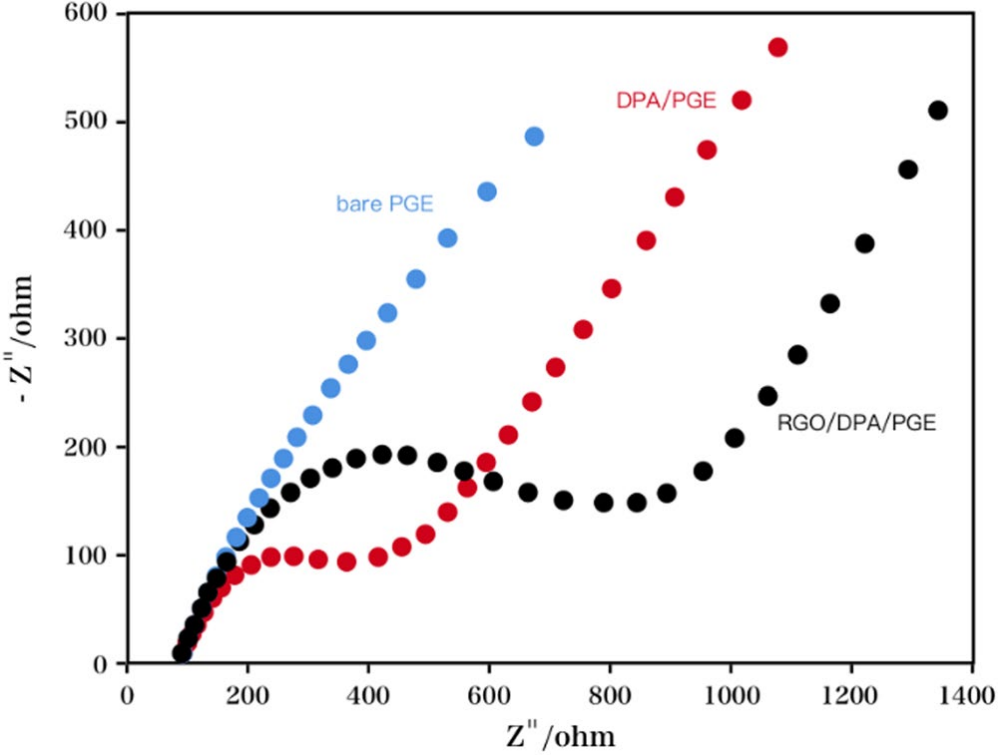
EIS responses of RGO/DPA/PGE, DPA/PGE, and bare PGE in 5.00 mM Fe(CN)_6_^4−/3−^ redox probe containing 0.10 M KCl solution.

For the measurement of the interface characteristics of the as-prepared electrodes, electrochemical impedance spectra (EIS) technique was used. The linear section at lower frequencies indicated the diffusional limited electron transfer process; while the semicircle section at higher frequencies represented the electron transfer limited process on the electrode interface, suggesting the charge transfer resistance (*R*_ct_). The impedance spectrum (Fig. 4) of bare PGE, DPA/PGE, and RGO/DPA/PGE was plotted in in 5.00 mM Fe(CN)_6_^4−/3−^ redox probe containing 0.10 M KCl solution. It can be seen that the lowest *R*_ct_ value (0.001 Ω) was shown at DPA/PGE, suggesting a desirable conductivity. An increase in *R*_ct_ value was observed at bare PGE (12.47 Ω), indicating a layer inhibiting the electron transfer between the Fe(CN)_6_^3−^/Fe(CN)_6_^4−^ system and PGE was formed. The maximal *R*_ct_ value (490.22 Ω) for the redox process was recorded after adding graphene to the composite. The RGO/DPA/PGE exhibited the largest Nyquist diameter among the other two electrodes. The *R*_ct_ value increased, possible due to the the hindrance of the electrostatic repulsion between the DPA and RGO on the modified electrode surface and the Fe(CN)_6_^4−/3−^ in the solution^36^. The increase in the semicircular diameter was observed at the RGO modified electrode, suggesting that a layer inhibiting the electron transfer from the redox probe [Fe(CN)_6_^4−/3−^] to the electrode surface was formed. The above results confirmed the surface modification of PGE using RGO and DPA.

**Figure 5:**
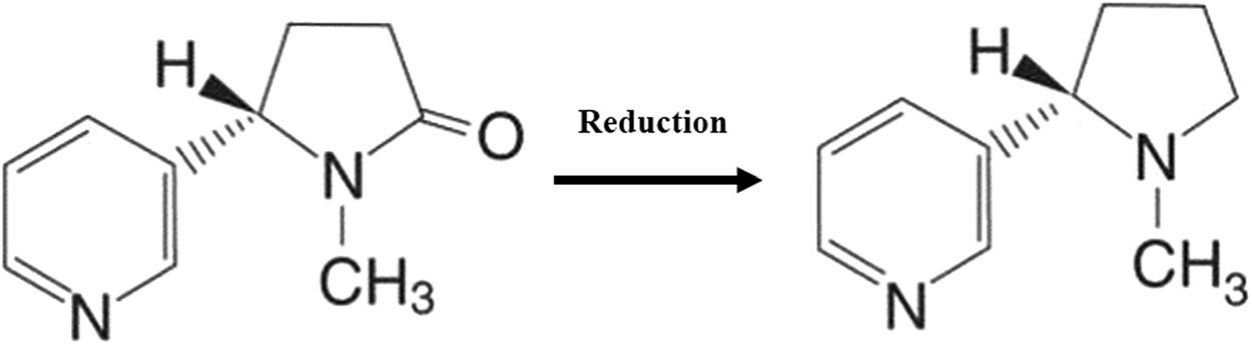
Schematic diagram of nicotine reduction process on an RGO/DPA/PGE.

The electroanalytical detection of nicotine or its primary metabolite, cotinine (Fig. 5), is not straightforward. Nicotine appears to display reversible electrode kinetics on most electrode surfaces, with any reductive features obscured by solvent breakdown or surface reduction. Therefore, we attempted to detecting nicotine based on RGO/DPA/PGE due to its outstanding electrochemical properties.

**Figure 6:**
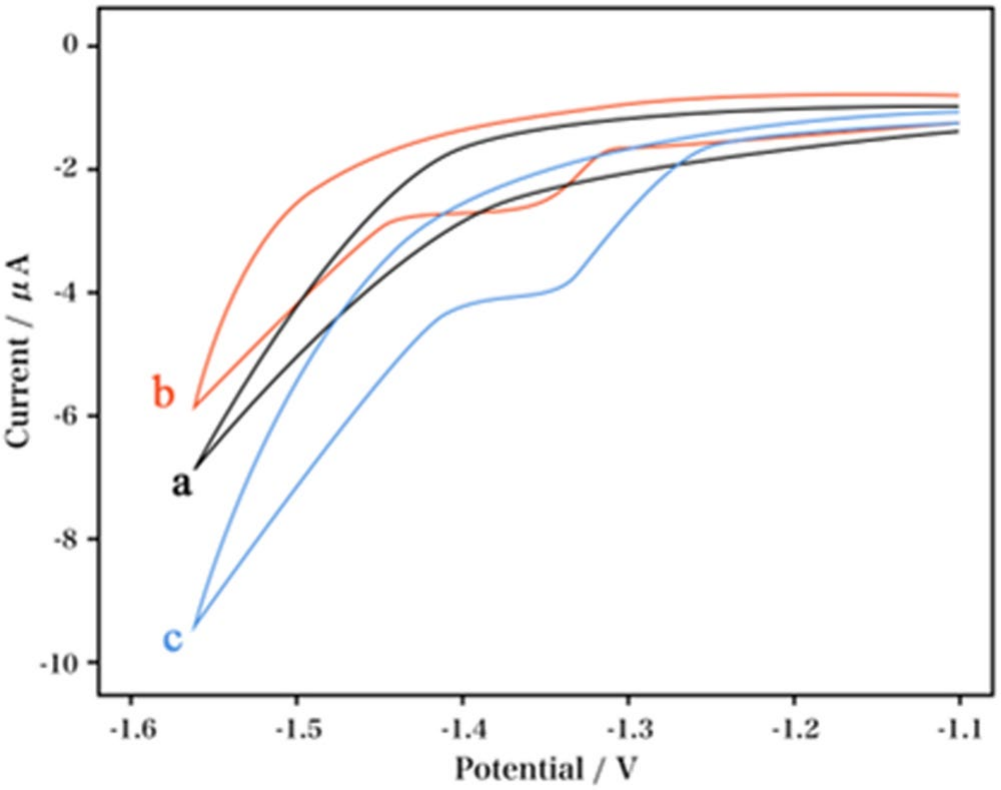
CVs obtained at the (a) bare PGE, (b) RGO/DPA/PGE without nicotine in Na_2_C_2_O_4_, (c) RGO/DPA/PGE with 0.3 mM nicotine in 0.1 M Na_2_C_2_O_4_ solution (pH 4.5).

Figure 6 showed the CVs of nicotine in Na_2_C_2_O_4_ solution (0.1 M; pH 4.5) using different electrodes. At the bare PGE and RGO/DPA/PGE, nicotine exhibited a reduction peak at *ca*. −1.4 V without oxidation peak, showing an irreversible property. At the RGO/DPA/PGE, nicotine showed an obviously increased current response, suggesting the effective modification of the bare electrode using MWNT. It can be seen that the RGO/DPA/PGE showed more desirable behavior, possibly ascribed to the topological defects and electronic structure on the surfaces of the RGO^21^. After several scanning cycles, a pronounced decrease in the current of nicotine was observed at the bare PGE, which was even disappearing. The reductive product adsorbed on the electrode surface was supposed to result in blunt electrode, and the blocking of further nicotine reduction. These results proved the inappropriateness of the bare electrode for the analysis of nicotine. No electrochemical response was shown at the modified electrode when without nicotine, indicating that the peak observed at −1.4 V resulted from the reduction of nicotine.

This report also optimized the buffer solution. The sensitivity was higher with fine nicotine voltammograms recorded in Na_2_C_2_O_4_ (0.1 M), compared with other supporting electrolytes including HAc-NaAc, H_2_SO_4_ and KH_2_PO_4_-NaHPO_4_ which were not suitable for the analysis of nicotine. In 0.1 M Na_2_C_2_O_4,_ insignificant peak of nicotine was observed at a pH of below 4, whereas reduction peak was absent at a pH of over 5. Therefore pH was optimized at a range of 4 to 5 for the investigation of the effect of pH on the response of nicotine. When the pH was 4.5, the maximum current was obtained, thus the pH of Na_2_C_2_O_4_ (0.1 M) was adjusted at 4.5 for the analysis of nicotine.

**Figure 7:**
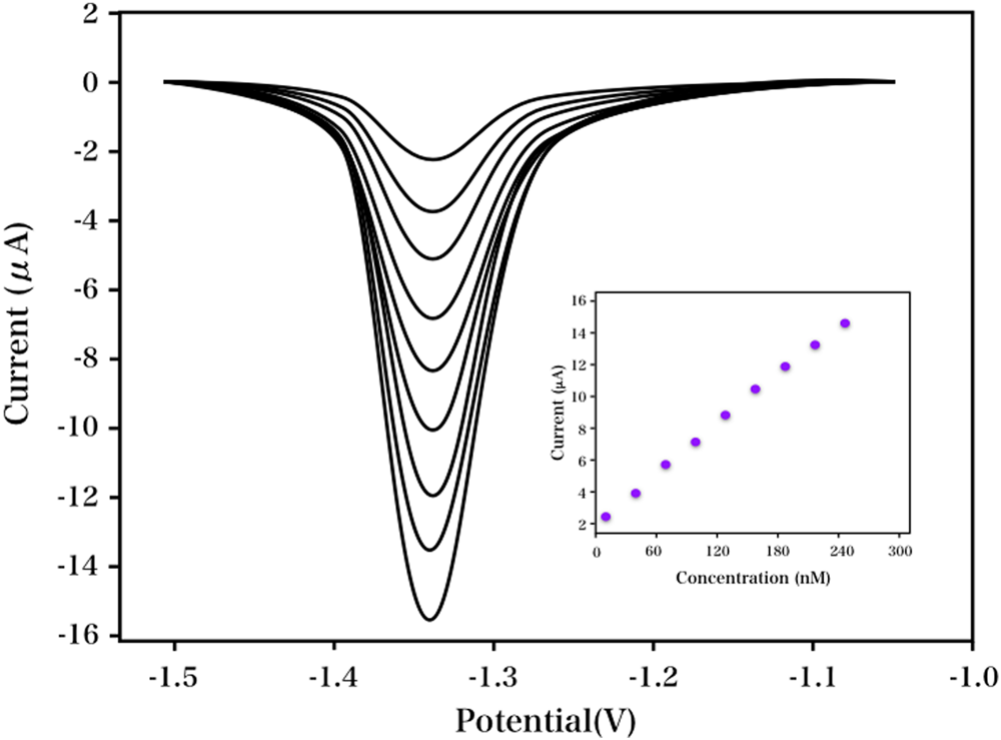
Baseline-corrected 1^st^ order derivative of LSVs of 0.3 mM nicotine in 0.1 M Na_2_C_2_O_4_ solution (pH 4.5) at different scan rate; Plots of reductive peak current versus scan rate (Inset).

Linear scan voltammetry (LSV) measurement was performed to study the effect of scan rate on the reduction of nicotine using RGO/DPA/PGE. Figure 7 and the inset plot showed that the peak current was in proportion to the scan rate (10–250 mV/s). It can be seen that the electrochemical performance of nicotine at RGO/DPA/PGE was an adsorption-controlled process.

**Figure 8:**
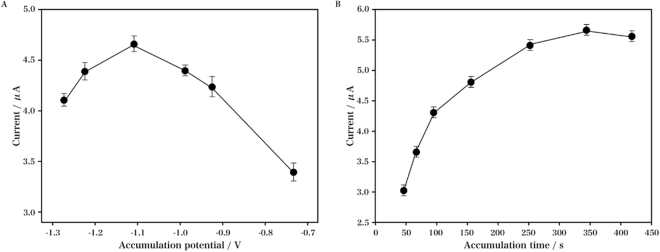
Effect of accumulation potential (**A**) and accumulation time (**B**) on the current of 0.3 mM nicotine in 0.1 M Na_2_C_2_O_4_ solution (pH 4.5).

The effect of accumulation potential on the response of nicotine (0.3 mM) was studied in a range of −0.7 to −1.3 V. Within a 2 min accumulation time, the peak current was increased with the potential decrease from −0.7 V, with the maximum peak current obtained at −1.1 V. Then the current was decreased when the potential exceeded −1.2 V. Hence the optimal accumulation potential was determined as −1.1 V. Another factor greatly affecting the peak current was the accumulation time. The measurement was performed at an accumulation potential of −1.1 V with different accumulation time ranging from 0 to 400 s. As shown in Fig. 8, the peak current was increased, with the maximum value obtained at *ca*. 250 s, indicating that the amount of nicotine adsorbed at the RGO/DPA/PGE surface showed a tendency of reaching saturation at an accumulation time of over 250 s. Based on the results of work efficiency and sensitivity, the optimum accumulation time for the analysis of nicotine was determined as 250 s.

**Figure 9:**
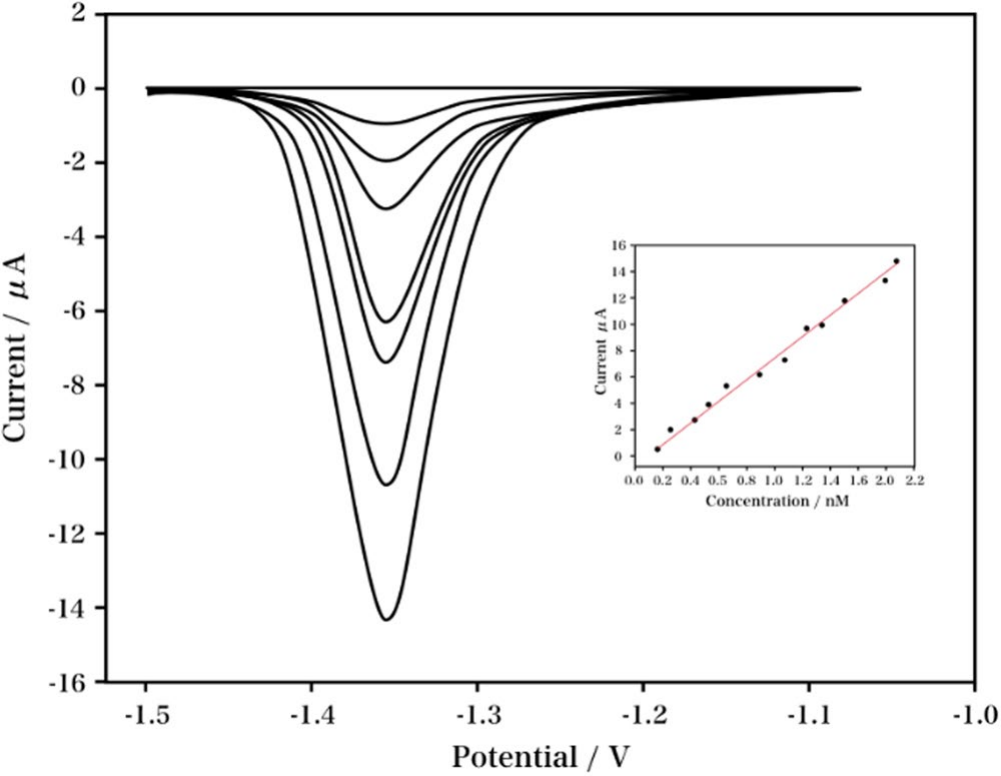
DPVs of different concentrations of nicotine in 0.1 M Na_2_C_2_O_4_solution (pH 4.5). Plots of reductive current versus the nicotine concentration (Inset).

The relationship between the nicotine concentration and current was investigated using differential pulse voltammetry (DPV) under optimum condition. As indicated in Fig. 9, the current was in proportion to the nicotine concentration over two linear ranges: from 31 to 1900 μM. The linear range was wider than the valves of 7.6 µM. The relative standard deviation (RSD) of the nicotine using a single modified electrode was obtained as 2.71%, indicating the proposed electrode was highly reproducible. To allow for comparison to previous reports, the characteristics of different electrochemical sensors for nicotine are summarized in Table 1.

**Table 1:** Comparison of the major characteristics of electrochemical sensors used for the detection of nicotine.

Electrode	Linear detection range	Detection limit	Reference
Nitrogen-doped graphene/GCE	0 to 200 μM	0.27 μM	37
PoPD/GCE	0.000183 to 1.01 μM	55 pM	38
Boron-doped diamond electrode	0.5 to 200 μM	0.3 µM	30
RGO/DPA/PGE	31 to 1900 μM	7.6 µM	This work

**Figure 10:**
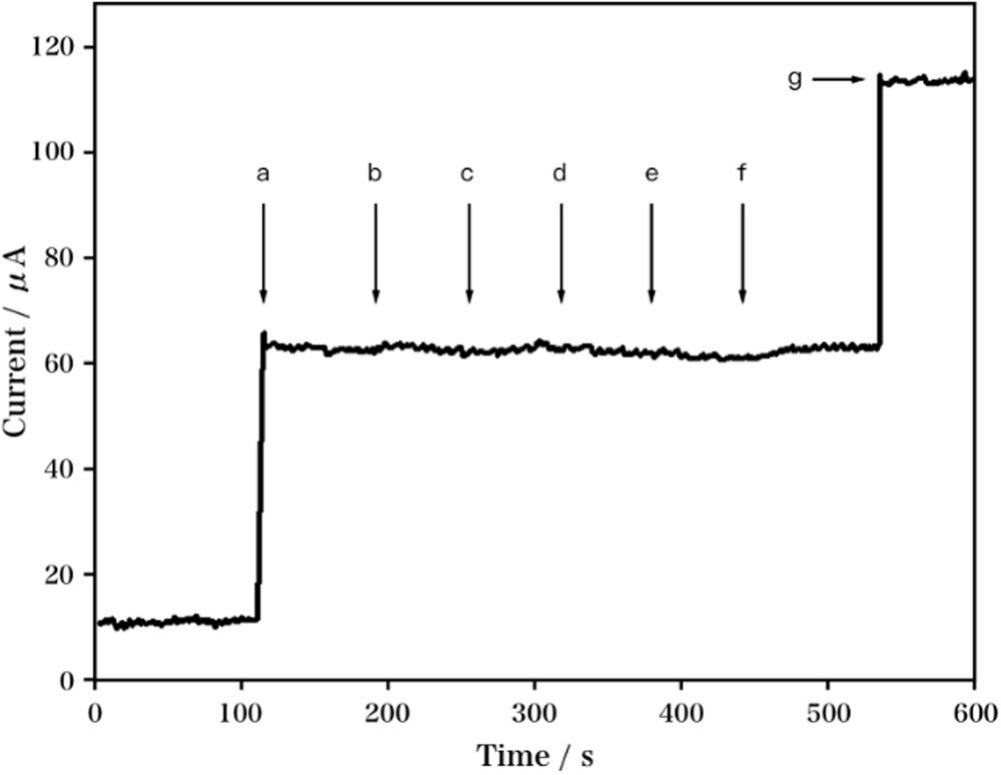
Amperometric current response of RGO/DPA/PGE after adding 50 μM nicotine (**a**), 1 mM cotinine (**b**), 1 mM ascorbic acid (**c**), 1 mM uric acid (**d**), 1 mM dopamine (**e**), 1 mM H_2_O_2_ (**f**) and 50 μM nicotine (**g**). Operating potential: 0.93 V.

It has been proposed that cotinine is a main metabolite of nicotine in urine and blood of human body. The effect of cotinine and several physiological interfering species were investigated so as to analyze the selectivity of our developed sensor. The characteristic amperometric response of RGO/DPA/PGE after adding nicotine, as well as several possible interfering substances such as H_2_O_2_, dopamine, uric acid, ascorbic acid, and cotinine was shown in Fig. 10. No significant variation was observed in the current response after adding H_2_O_2_, dopamine, uric acid, ascorbic acid, and cotinine (1 mM), suggesting the high selectivity of our developed sensor to the detection of nicotine, even after adding 20-fold excess of common interfering substances.

The content of nicotine in tobacco products were determined so as to study the practical use of the as-prepared RGO/DPA/PGE. In specific, two brands of cigarette and one cigar were selected to evaluate the feasibility of our developed sensor. The real sample test was performed using the standard addition method. The comparison of our developed RGO/DPA/PGE and a reference method (RP-HPLC)^39^ was presented in Table 2. These results showed that our developed RGO/DPA/PGE performed well in the electrochemical sensing to the determination of nicotine in commercial tobacco products, suggesting the potential of this electrode to be used for the determination of nicotine content in the real samples.

**Table 2:** Content analysis of the nicotine in two brands of cigarettes and pharmaceuticals using RGO/DPA/PGE.

Sample	Addition (μM)	Found (μM)	RSD (%)	Recovery (%)	RP-HPLC	RSD (%)
Cigarette 1	0	9.78	2.68	—	9.95	1.65
	20	29.89	3.62	100.37	29.96	2.09
	50	60.25	1.25	100.79	60.22	1.08
Cigarette 2	0	4.33	3.22	—	4.42	2.36
	20	23.96	1.54	98.48	22.54	2.07
	50	58.58	1.26	98.00	59.06	1.05
Cigar	0	15.26	3.26	—	14.26	5.33
	20	35.59	2.75	100.96	35.00	2.89
	50	66.36	3.21	101.69	53.11	3.01

## Conclusion

In this work, the RGO/DPA nanocomposite was prepared using a facile and mild route proposed herein. Based on the results of CV and EIS measurements, the developed RGO/DPA/PGE owns a desirable electrocatalytic activity to the detection of nicotine. Therefore this electrode was used for the fabrication of an electrochemical sensor towards the detection of nicotine with high sensitivity, selectivity and reliability. Our developed nicotine sensor had a wide linear response range of 31 to 1900 μM, as well as a low LOD of 7.6 μM. Furthermore, the developed nicotine sensor could be successfully used for the content analysis of nicotine in the tobacco product.
